# A Case Report on Distinguishing Emphysematous Pyelitis and Pyelonephritis on Point-of-care Ultrasound

**DOI:** 10.5811/cpcem.2020.11.49892

**Published:** 2020-12-31

**Authors:** Proma Mazumder, Fares Al-Khouja, John Moeller, Shadi Lahham

**Affiliations:** * Touro University Nevada, School of Osteopathic Medicine, Henderson, Nevada; † University of California, Irvine, School of Medicine, Irvine, California; ‡ University of California, Irvine, Department of Emergency Medicine, Orange, California

**Keywords:** Point-of-care ultrasound, emphysematous pyelitis, emphysematous pyelonephritis

## Abstract

**Introduction:**

Point-of-care ultrasound (POCUS) in the emergency department (ED) is being performed with increasing frequency. The objective of this study was to demonstrate how utilization of POCUS can help the emergency physician recognize emphysematous pyelitis (EP) and emphysematous pyelonephritis (EPN).

**Case Report:**

A 60-year-old female presented to the ED with normal vital signs and intermittent left-sided flank pain that radiated to her groin. She also had a history of obstructive nephrolithiasis. Within 20 minutes of arrival she became febrile (101.2°Fahrenheit), tachycardic (114 beats per minute), tachypneic (21 breaths per minute), and had a blood pressure of 114/82 millimeters mercury. POCUS was conducted revealing heterogeneous artifact with “dirty shadowing” within the renal pelvis, which was strongly suggestive of air. The emergency physician ordered a computed tomography (CT) to confirm the suspicion for EP and started the patient on broad-spectrum antibiotics. The CT showed a 1.3-centimeter calculus and hydronephrosis with foci of air. The patient received intravenous antibiotics and had an emergent nephrostomy tube placed. Urine cultures tested positive for pan-sensitive *Escherichia Coli*. Urology was consulted and a repeat CT was obtained to show correct drainage and decreased renal pelvis dilation.

**Conclusion:**

Distinctly different forms of treatment are used for EP and EPN, despite both having similar pathophysiology. In EP, air can be seen in the renal pelvis on POCUS, as in this case study, which distinguishes it from EPN. In the case of our patient, the use of POCUS was useful to aid in rapid differentiation between EP and EPN.

## INTRODUCTION

Emphysematous pyelitis (EP) is a rare complication of pyelonephritis that results from gas-forming bacteria localized to the renal pelvis or renal collecting system.^1^ Emphysematous pyelitis is a relatively benign condition when compared to emphysematous pyelonephritis (EPN). While EP involves an infection of the renal pelvis by gas-forming bacteria, EPN consists of a necrotizing infection of the renal parenchyma as well. Both EP and EPN are rare complications of acute pyelonephritis. However, EPN can have devastating outcomes with mortality rates as high as 80% if treated with antibiotics alone.^2^ The clinical presentation of both entities is remarkably similar, consisting of fever, chills, flank pain, dysuria, vomiting, and lethargy.^2^

Historically, the only method of differentiation between EP and EPN has been computed tomography (CT) that demonstrates air within the renal parenchyma. It is important to distinguish EPN from EP due to the increased morbidity and mortality associated with EPN, as well as the different treatment course for each condition.^2^,^3^ In this report, we demonstrate the utility of point-of-care ultrasound (POCUS) to diagnose EP in a female presenting with symptoms suggestive of pyelonephritis.

## CASE REPORT

A 60-year-old female with an extensive history of obstructive nephrolithiasis presented to our emergency department (ED) with left-sided flank pain. She described her pain as intermittent and sharp in nature, with radiating pain to the groin. Associated complaints included dysuria and gross hematuria. Initial triage vitals were normal; however, within 20 minutes of arrival she became febrile to 101.2° Fahrenheit, tachycardic to 114 beats per minute, tachypneic to 21 breaths per minute, and a blood pressure of 114/82 milligrams mercury. On exam she was in mild distress, with diaphoresis and left costovertebral angle tenderness.

Point-of-care ultrasound performed in the ED showed unilateral moderate hydronephrosis with echogenic debris in the renal pelvis (Video). Specifically, the isolated debris in the renal pelvis was heterogeneous with both hyperechoic and isoechoic artifacts; mobile hyperechoic foci with “dirty shadowing” were highly suggestive of air in the renal pelvis. Blood cultures were obtained, and given suspicion for EP, the emergency physician initiated broad-spectrum antibiotics and consulted urology while waiting for the results of a confirmatory CT.

The CT demonstrated a 1.3-centimeter calculus and hydronephrosis with foci of air, raising suspicion for a hemorrhagic or infectious etiology (Image). The patient was admitted for intravenous antibiotics and emergent nephrostomy tube placement by interventional radiology. Blood cultures were positive for gram-negative rods. Urine cultures revealed pan-sensitive *Escherichia coli*. The patient was evaluated by the urology service, with repeat CT showing appropriate drainage of infection and decreased renal pelvis dilation.

**CPC-EM Capsule**

What do we already know about this clinical entity?

Emphysematous pyelitis (EP) and emphysematous pyelonephritis (EPN) are diagnosed through computed tomography (CT); treatment differs despite similar pathophysiology.

What makes this presentation of disease reportable?

The ultrasound video clip demonstrates key findings such as reverberation artifact and “dirty shadowing” to show EP.

What is the major learning point?

Ultrasound may be useful to differentiate pathology for EP and EPN.

How might this improve emergency medicine practice?

Using ultrasound may expedite diagnosis of EP and EPN to better guide course of therapy before confirming with CT.

## DISCUSSION

Emphysematous infections of the renal and genitourinary collecting systems can be life threatening and rapidly progress to sepsis without aggressive intervention.^4^ Emphysematous pyelonephritis is characterized by a necrotic infection of the renal parenchyma. Infection with gas-forming microbes will result in the presence of gas in the collecting system and perinephrotic tissue.^5^ Acute EPN can result in greater complications then EP due to the increased rate of sepsis. The primary cause of mortality in EPN is complications related to sepsis.^5^

Although both EP and EPN have similar pathophysiology and epidemiological risk factors, the overall prognosis of each pathology and diagnostic criteria are unique. Specifically, EP has a significantly lower mortality rate (18–20%) as compared to the nearly twofold increase in mortality associated with EPN (25–42%). 4,6,7 The clinical presentation of EP can be similar to that of pyelonephritis, with symptoms ranging from fever, chills, hematuria, and vomiting to renal angle tenderness.^1^ In contrast, the presentation of EPN is typically more ominous, frequently presenting with vital sign abnormalities, sepsis, and shock if left untreated.^8^

Computed tomography is considered the best modality for differentiating EPN from EP, as it can consistently differentiate the existence of gas in specific locations within the renal excretory system, including renal parenchyma, renal pelvis, and perinephric spaces.^3^ Although there have been a few case reports of the adjunctive roles of ultrasound and kidney, ureter, and bladder radiographs in differentiating EP from EPN, to our knowledge the reported use of POCUS by an emergency physician for rapid identification of EP is unique. Given routine delays in CT imaging in a busy ED, the role of POCUS in helping physicians differentiate between a benign and life-threatening condition has evolved.

POCUS for both EP and EPN demonstrates a reverberation artifact projecting posteriorly from a hyperechoic focus (emphysema). The hyperechoic focus is due to free air bubbles with lateral and axial blooming.^9^ The reverberation artifact from the hyperechoic gas bubbles gives a sonographic appearance of “dirty shadowing.” Dirty shadowing is described as superimposed echoes from free gas that give a large radius of curvature of the surface struck by the sound beam.^10^ This results in the characteristic dirty shadowing that projects from a hyperechoic focus of free gas with a larger curvature and acoustic noise within the shadow. In contrast, “clean shadowing” is related to solid surface material, such as nephroliths, that creates a clean shadow with no infiltrative artifact.^10^ Characteristics of EP on POCUS that distinguish it from EPN include the presence of “dirty shadowing,” which is isolated to the renal pelvis.^11^ In comparison, sonographic findings of EPN are similar; however, reverberation artifact is present extending toward the renal parenchyma, not isolated to the renal pelvis as in EP. At times, emphysema into the parenchyma and renal cortex can become so extensive that adequate visualization of the pelvis and uropelvic junction can be challenging.^12^

Current treatment of EP consists of the use of broad-spectrum antibiotics, urology evaluation, and nephrostomy tube placement in cases of obstructive processes.^13^ In contrast, EPN requires aggressive interventions. Delays in recognition and/or diagnosis by the provider can result in increased morbidity or mortality. POCUS can help facilitate the prompt diagnosis and treatment of both EP and EPN, resulting in improved patient outcomes.^1^,^2^,^14^

## CONCLUSION

Point-of-care ultrasound can be used successfully by emergency physicians to rapidly differentiate between emphysematous renal infections, thus expediting care in critically ill patients. This case report further characterizes the sonographic appearance of emphysematous pyelitis, as well as comparing the subtle differences in ultrasound imaging, presentation, and treatment of EP from the far deadlier emphysematous pyelonephritis.

## Supplementary Information

Video. This video clip shows a coronal ultrasound of the left kidney presented in the case report. As seen, there is an obvious hypoechoic dilation of the renal pelvis with blunting of the calyces consistent with moderate hydronephrosis. Also visualized in the clip is a heterogeneous collection of debris. The isoechoic sediment likely represents purulent material in the clinical setting of infection. The hyperechoic foci, with posterior “dirty shadowing,” corresponds to air. The foci of air are isolated to the renal pelvis consistent with the diagnosis of emphysematous pyelitis.

## Figures and Tables

**Image:**
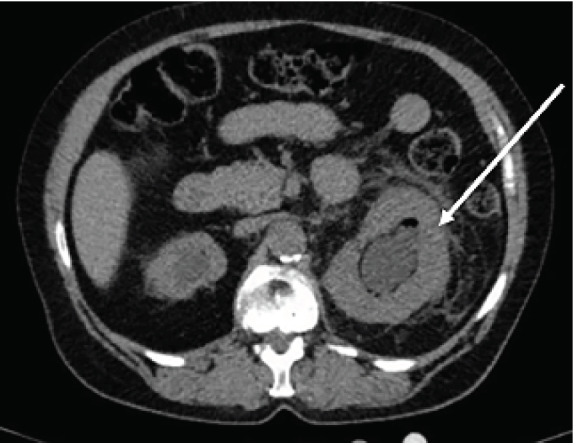
Computed tomography of the abdomen and pelvis showing an enlarged left kidney with hydronephrosis as well as air in the renal pelvis (white arrow).
